# Pain outcomes in emergent versus elective ventral hernia repair: Propensity score matched analysis

**DOI:** 10.1007/s10029-026-03842-0

**Published:** 2026-10-01

**Authors:** Sidhant Kalsotra, Li-Ching Huang, Savannah Renshaw, Benjamin K. Poulose

**Affiliations:** 1 Center for Abdominal Core Health, Department of Surgery, The Ohio State University Wexner, Columbus, OH USA; 2 Medical Center, Columbus, OH USA; 3 Department of Surgery, The Ohio State University Wexner Medical Center, Doan Hall N729, 410 W. 10th St., Columbus, OH 43210 USA; 4 Department of Biostatistics, Vanderbilt University Medical Center, Nashville, TN USA

**Keywords:** Ventral hernia repair, Emergent surgery, Patient-reported outcomes, PROMIS, Pain

## Abstract

**Introduction:**

Whether emergent ventral hernia repair (VHR) compromises long-term patient-reported pain recovery compared to elective repair remains unknown. We compared Patient-Reported Outcomes Measurement Information System Pain Intensity (PROMIS Pain 3a) outcomes between emergent and elective VHR using a multicenter registry after risk adjustment.

**Methods:**

We performed a retrospective cohort analysis using the Abdominal Core Health Quality Collaborative (ACHQC) registry of adults undergoing VHR from 2013 to 2024 with complete baseline and 30-day PROMIS Pain 3a scores were included. Emergent and elective repairs were compared using 1:3 propensity score matching (PSM) across 31 covariates. The primary outcome was PROMIS Pain 3a T-score at one year postoperatively.

**Results:**

Of 11,831 patients (94 emergent, 11,737 elective), emergent patients had higher baseline PROMIS Pain 3a T-scores (median 52 [IQR 46–58] vs. 44 [IQR 36–52], *p* < 0.001), BMI (median 36 [IQR 32–41] vs. 31 [IQR 27–36] kg/m², *p* < 0.001), and ASA class 3–4 rates (66% vs. 55%, *p* < 0.001). After PSM (71 emergent, 193 elective), baseline pain scores were equivalent (median 52 [IQR 44–58] vs. 49 [IQR 44–54], *p* = 0.2). At 30 days, pain scores did not differ (median 44 [IQR 40–52] vs. 46 [IQR 40–52], *p* = 0.6). At one year, emergent patients reported significantly lower PROMIS Pain 3a T-scores (median 31 [IQR 31–38] vs. 40 [IQR 31–46], *p* = 0.01) and greater improvement from baseline (median − 21 [IQR − 24 to − 11] vs. −8 [IQR − 16 to 0], *p* = 0.002). Complication rates and pragmatic recurrence through five years were equivalent between matched groups.

**Conclusion:**

Emergent VHR patients demonstrated superior patient-reported pain outcomes at one year compared to propensity-matched elective controls, with equivalent complication and recurrence rates. These findings challenge the notion that emergent repairs inherently lead to worse outcomes and suggest that surgical urgency may not negatively impact long-term pain recovery.

**Supplementary Information:**

The online version contains supplementary material available at https://doi.org/10.1007/s10029-026-03842-0.

## Introduction

Emergent ventral hernia repair (VHR) is widely perceived to carry worse outcomes than elective repair, due to the acuity of presentation, higher rates of bowel obstruction, contaminated operative fields, and the physiologic burden of urgent surgery [1, 2]. While prior studies have examined short-term clinical outcomes such as wound complications, reoperation, and mortality in this population [2], patient-reported pain outcomes following emergent VHR remain largely uncharacterized. The Patient-Reported Outcomes Measurement Information System Pain Intensity (PROMIS Pain 3a) has emerged as a reasonable instrument for quantifying pain after VHR, particularly within the Abdominal Core Health Quality Collaborative (ACHQC), the largest prospective hernia registry in the United States, where a robust body of literature now reports PROMIS Pain 3a trajectories at 30 days and one year following elective repair across a range of operative approaches, mesh types, and patient populations [3–6]. Despite this growing evidence base, limited data exists on reported PROMIS Pain 3a outcomes stratified by repair acuity, representing a persistent and well-documented gap in the abdominal wall surgery literature [1, 7]. 

The ACHQC registry offers a unique opportunity to address this gap, capturing granular preoperative, intraoperative, and longitudinal patient-reported outcome data across a diverse, multicenter cohort of patients undergoing both emergent and elective VHR [1, 5, 6, 8–10]. Given the significant baseline differences between these populations, including higher pain burden, greater hernia complexity, and increased comorbidity in emergent patients, rigorous risk adjustment is essential to isolate the effect of surgical acuity on pain recovery [1, 2]. We therefore conducted a propensity score matched analysis of ACHQC registry data to test the hypothesis that emergent VHR is associated with worse long-term patient-reported pain outcomes compared to elective repair.

## Methods

### Design overview

We conducted a retrospective cohort analysis using the Abdominal Core Health Quality Collaborative (ACHQC) registry from January 2013 through December 2024. The ACHQC is a prospectively maintained, multicenter quality improvement registry capturing patient demographics, operative details, and patient-reported outcomes (PROs) for abdominal core health procedures across participating institutions in the United States [11]. Emergent and elective ventral hernia repair (VHR) were compared using propensity score matching (PSM) to evaluate differences in postoperative pain outcomes. This study was approved by the Ohio state institutional review board #2024E0086.

### Study population and data source

Adult patients who underwent open, laparoscopic, or robotic VHR with complete baseline and 30-day patient-reported pain outcomes were eligible for inclusion. Patients with missing data for surgical setting (emergent vs. elective) were excluded, yielding a final analytical cohort of 11,831 patients.

#### Comparison groups

The exposure variable was surgical setting, defined as emergent or elective VHR as recorded by the operating surgeon at the time of case entry into the ACHQC registry. Emergent repair was defined as surgery performed for acute indications requiring unplanned, urgent operative intervention, including but not limited to bowel obstruction, incarceration, or strangulation. Elective repair encompassed all planned, non-urgent procedures. Of 11,831 patients meeting inclusion criteria, 94 underwent emergent repair and 11,737 underwent elective repair.

### Outcome measures

The primary outcome was the PROMIS Pain 3a T-score at one year postoperatively. The PROMIS Pain 3a consists of three questions assessing pain intensity over the preceding seven days, with raw scores converted to T-scores standardized to the United States general population (mean 50, standard deviation 10), where higher scores indicate greater pain intensity. Secondary outcomes included PROMIS Pain 3a scores at 30 days, change in PROMIS Pain 3a from baseline at each time point, Hernia-Related Quality of Life Survey (HerQLes) scores, 30-day surgical site infection (SSI), surgical site occurrence (SSO), readmission, reoperation, and clinical hernia recurrence.

### Statistical analysis

Continuous variables were summarized as medians with interquartile ranges (IQR) and compared using Wilcoxon rank-sum tests; categorical variables were summarized as frequencies with proportions and compared using Pearson chi-square tests. To account for baseline differences between emergent and elective patients, PSM was performed at a 1:3 ratio (emergent: elective) using a nearest-neighbor algorithm with a caliper width of 0.2 standard deviations of the logit of the propensity score. The PSM model included 31 covariates encompassing patient demographics, comorbidities, hernia characteristics, operative details, and baseline PRO scores, including baseline PROMIS Pain 3a and HerQLes. A total of 418 patients were excluded from PSM due to missing covariate data, yielding a matched cohort of 264 patients (71 emergent, 193 elective). Balance between matched groups was assessed using standardized mean differences (SMD), with SMD < 0.20 considered acceptable balance. Statistical significance was set as two-sided *p* < 0.05 All analyses were performed using R version 4.5.

## Results

### Study population

A total of 11,831 patients met inclusion criteria, of whom 94 underwent emergent VHR and 11,737 underwent elective VHR. Baseline demographics, hernia characteristics, and operative details (Table 1). Mean age was similar between groups (emergent 57 ± 13 years vs. elective 57 ± 13 years, *p* = 0.81). Emergent patients had a higher mean BMI (37 ± 8 vs. 32 ± 7 kg/m², *p* < 0.001), higher rates of ASA class 3 or 4 designation (66% vs. 55%, *p* < 0.001), and greater rates of recurrent hernia (38% vs. 27%, *p* = 0.02). Emergent repairs more frequently involved contaminated or dirty wound classifications (17% vs. 5%, *p* < 0.001), higher-grade hernias (Grade 3: 32% vs. 12%, *p* < 0.001), and bowel obstruction as the primary indication for surgery (54% vs. 5%, *p* < 0.001). Mesh was used less frequently in emergent cases (80% vs. 87%, *p* = 0.05). Median length of hospital stay was longer in the emergent group (3 days vs. 1 day, *p* < 0.001). Baseline PROMIS Pain 3a T-scores and HerQLes summary scores, reflect the greater preoperative pain burden and lower health-related quality of life in the emergent cohort (Table 1).

**Table 1:** Baseline demographics and operative characteristics (Unmatched cohort)

Characteristic	Emergent VHR (*n* = 94)	Elective VHR (*n* = 11,737)	*P*-value
Demographics			
Age, median (IQR) (years)	58 (48–65)	58 (47–67)	0.81
Female sex, n (%)	53 (56%)	5,451 (46%)	0.052
BMI, median (IQR) (kg/m²)	36 (32–41)	31 (27–36)	< 0.001
White race, n (%)	79 (84%)	10,187 (87%)	0.82
Hypertension, n (%)	56 (60%)	5,537 (47%)	0.022
Diabetes mellitus, n (%)	22 (23%)	2,030 (17%)	0.12
Current smoker, n (%)	8 (9%)	1,007 (9%)	0.12
Dialysis, n (%)	2 (2%)	62 (1%)	0.042
Dyspnea, n (%)	6 (6%)	261 (2%)	0.007
History of IBD, n (%)	8 (9%)	441 (4%)	0.02
ASA Class 3–4, n (%)	62 (66%)	6,362 (55%)	< 0.001
Hernia Characteristics			
Recurrent hernia, n (%)	36 (38%)	3,214 (27%)	0.02
Hernia grade 3, n (%)	30 (32%)	1,389 (12%)	< 0.001
Incisional hernia, n (%)	62 (66%)	7,340 (63%)	0.51
Umbilical hernia, n (%)	23 (24%)	3,525 (30%)	0.21
Parastomal hernia, n (%)	11 (12%)	670 (6%)	0.003
Stoma present, n (%)	11 (12%)	723 (6%)	0.03
Hernia width, median IQR (cm)	5 (2–8)	5 (2–11)	0.92
Operative Details			
Open approach, n (%)	73 (78%)	8,276 (71%)	0.11
Laparoscopic approach, n (%)	8 (9%)	806 (7%)	
Robotic approach, n (%)	13 (14%)	2,655 (23%)	
Wound class contaminated/dirty, n (%)	16 (17%)	523 (5%)	< 0.001
Bowel obstruction as indication, n (%)	51 (54%)	575 (5%)	< 0.001
Mesh used, n (%)	75 (80%)	10,155 (87%)	0.05
Myofascial release, n (%)	33 (35%)	5,122 (44%)	0.11
OR time > 2 h, n (%)	46 (49%)	5,437 (46%)	0.61
Length of stay, median IQR (days)	3 (0–4)	1 (0–4)	< 0.001
Baseline Patient-Reported Outcomes			
PROMIS Pain 3a T-score, median (IQR)	52 (46–58)	44 (36–52)	< 0.001
HerQLes summary score, median (IQR)	40 (22–67)	48 (27–73)	0.03

*BMI* body mass index, *IBD* inflammatory bowel disease, *ASA* American Society of Anesthesiologists, *PROMIS* Patient-Reported Outcomes Measurement Information System, *HerQLes* Hernia-Related Quality of Life Survey, *IQR* interquartile range, *SD* standard deviation

### Propensity score matched outcomes

**Fig. 1:**
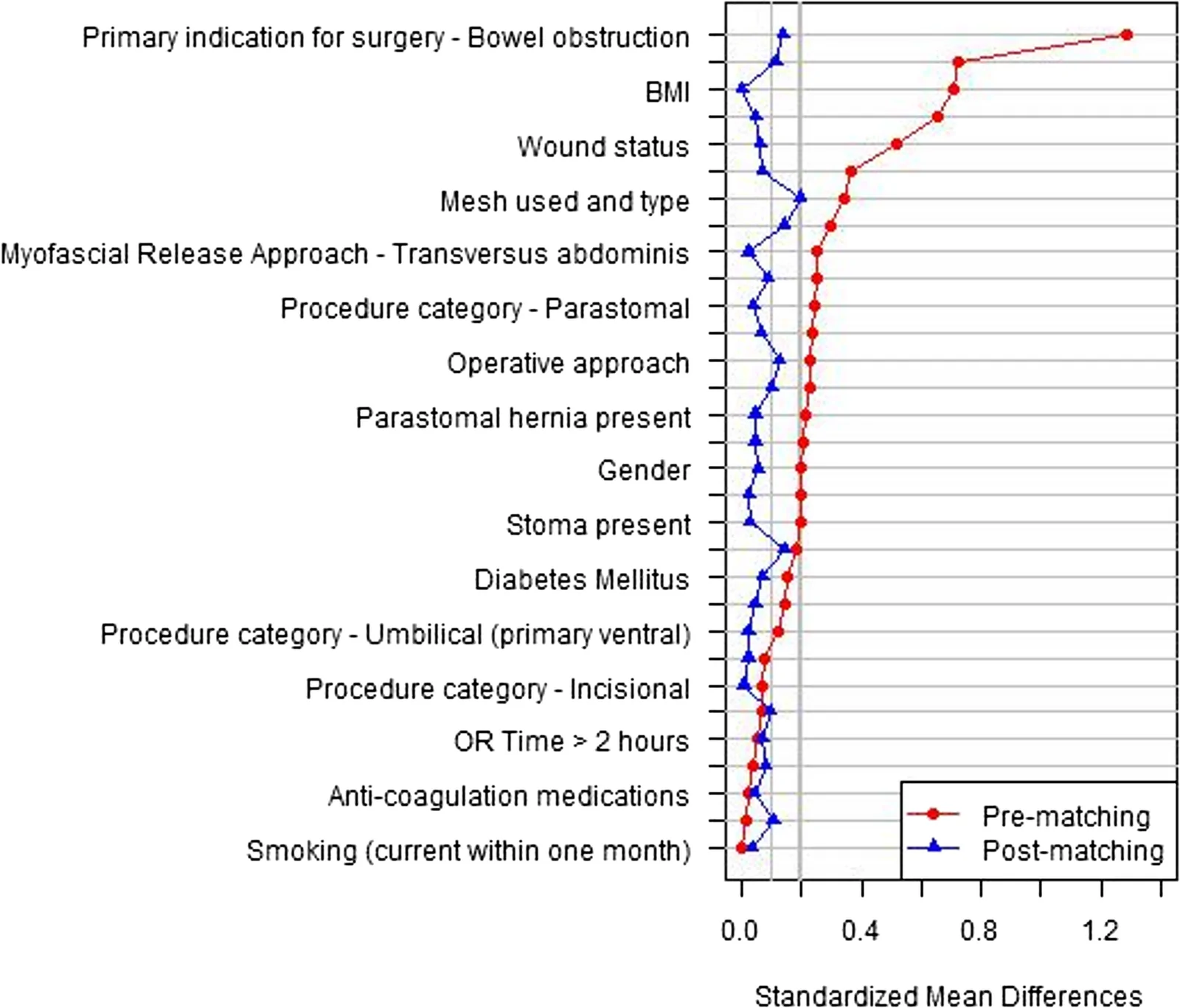
Standardized mean difference (SMD) love plot demonstrating covariate balance before (red circles) and after (blue triangles) propensity score matching. Each point represents one of 31 covariates included in the propensity score model. Post-matching SMD values below 0.20 across all covariates indicate adequate balance between the emergent and elective ventral hernia repair groups. SMD, standardized mean difference

After propensity score matching, 264 patients were included in the matched cohort (71 emergent, 193 elective). Adequate covariate balance was achieved across all matched variables, as demonstrated by standardized mean differences below 0.20 (Fig. 1). Post-matching baseline characteristics, 30-day surgical outcomes, and hernia recurrence data are presented in Table 2. Baseline PROMIS Pain 3a scores were similar between matched groups (emergent median 52 [IQR 44–58] vs. elective median 49 [IQR 44–54], *p* = 0.2). At 30 days, pain scores remained equivalent (emergent median 44 [IQR 40–52] vs. elective median 46 [IQR 40–52], *p* = 0.6), and the change from baseline did not differ significantly (median − 5.4 [IQR − 11.1 to 0.0] vs. −2.7 [IQR − 8.2 to 2.4], *p* = 0.1). At one year, emergent patients reported significantly lower PROMIS Pain 3a T-scores than elective patients (median 31 [IQR 31–38] vs. 40 [IQR 31–46], *p* = 0.01), corresponding to a greater improvement from baseline (median − 21 [IQR − 24 to − 11] vs. −8 [IQR − 16 to 0], *p* = 0.002) (Table 3). Among 30-day clinical outcomes after matching, rates of SSI (3% vs. 3%, *p* = 0.9), SSO (12% vs. 7%, *p* = 0.2), readmission (5% vs. 3%, *p* = 0.7), reoperation (0% vs. 2%, *p* = 0.3), and overall complications (21% vs. 13%, *p* = 0.1) did not differ significantly between groups (Table 2). Pragmatic recurrence rates through five years of follow-up were comparable between matched groups at all time points, with no statistically significant differences observed (Table 2).

**Table 2:** Baseline balance, 30-day surgical outcomes, and hernia recurrence — propensity score matched cohort (Emergent *n* = 71, Elective *n* = 193)

	Emergent VHR	Elective VHR	*P*-value
Baseline Characteristics After Matching			
Age, median (IQR) (years)	55 (46–64)	53 (45–63)	0.31
Female sex, n (%)	37 (52%)	106 (55%)	0.72
BMI, median (IQR) (kg/m²)	36 (30–40)	35 (30–42)	0.82
ASA Class 3–4, n (%)	47 (66%)	122 (63%)	1.0
Wound class contaminated/dirty, n (%)	8 (11%)	22 (11%)	1.0
Bowel obstruction as indication, n (%)	34 (48%)	79 (41%)	0.31
Mesh used, n (%)	59 (83%)	163 (84%)	0.81
PROMIS Pain 3a T-score at baseline, median (IQR)	52 (44–58)	49 (44–54)	0.20
HerQLes score at baseline, median (IQR)	40 (22–62)	45 (25–65)	0.40
30-Day Surgical Outcomes			
SSI, n (%)	2 (3%)	6 (3%)	0.91
SSO, n (%)	8 (12%)	12 (7%)	0.21
Readmission, n (%)	3 (5%)	6 (3%)	0.71
Reoperation, n (%)	0 (0%)	3 (2%)	0.31
Any complication, n (%)	15 (21%)	26 (13%)	0.11
Hernia Recurrence			
Pragmatic recurrence at 1 year, n/N (%)	3/26 (12%)	11/85 (13%)	0.80
Pragmatic recurrence at 2 years, n/N (%)	1/18 (6%)	11/61 (18%)	0.20
Pragmatic recurrence within 5 years, n/N (%)	3/26 (12%)	23/91 (25%)	0.10

*SSI* surgical site infection, *SSO* surgical site occurrence, *PROMIS* Patient-Reported Outcomes Measurement Information System, *HerQLes* Hernia-Related Quality of Life Survey, *SD* standard deviation

### Extended follow-up and secondary quality-of-life outcomes

After propensity score matching, 264 patients were included in the matched cohort (71 emergent, 193 elective). Adequate covariate balance was achieved across all matched variables, as demonstrated by standardized mean differences below 0.20 (Fig. 1). Post-matching baseline characteristics, 30-day surgical outcomes, and hernia recurrence data are presented in Table 2. Baseline PROMIS Pain 3a scores were similar between matched groups (emergent median 52 [IQR 44–58] vs. elective median 49 [IQR 44–54], *p* = 0.2). At 30 days, pain scores remained equivalent (emergent median 44 [IQR 40–52] vs. elective median 46 [IQR 40–52], *p* = 0.6), and the change from baseline did not differ significantly (median − 5.4 [IQR − 11.1 to 0.0] vs. −2.7 [IQR − 8.2 to 2.4], *p* = 0.1). At one year, emergent patients reported significantly lower PROMIS Pain 3a T-scores than elective patients (median 31 [IQR 31–38] vs. 40 [IQR 31–46], *p* = 0.01), corresponding to a greater improvement from baseline (median − 21 [IQR − 24 to − 11] vs. −8 [IQR − 16 to 0], *p* = 0.002) (Table 3). Among 30-day clinical outcomes after matching, rates of SSI (3% vs. 3%, *p* = 0.9), SSO (12% vs. 7%, *p* = 0.2), readmission (5% vs. 3%, *p* = 0.7), reoperation (0% vs. 2%, *p* = 0.3), and overall complications (21% vs. 13%, *p* = 0.1) did not differ significantly between groups (Table 2). Pragmatic recurrence rates through five years of follow-up were comparable between matched groups at all time points, with no statistically significant differences observed (Table 3).

**Table 3:** Patient-reported pain and quality-of-life outcomes - propensity score matched cohort (Emergent *n* = 71, Elective *n* = 193)

Outcome	Emergent VHR	Elective VHR	*P*-value
PROMIS Pain 3a T-score, median (IQR)			
Baseline	52 (44–58)	49 (44–54)	0.20
30 days	44 (40–52)	46 (40–52)	0.60
1 year	31 (31–38)	40 (31–46)	**0.01**
2 years	31 (31–40)	40 (31–51)	0.10
3 years	40 (32–44)	40 (31–49)	0.60
4 years	31 (31–44)	31 (31–52)	0.80
5 years	31 (31–37)	31 (31–35)	0.90
Change in PROMIS Pain 3a from Baseline, median (IQR)			
30 days	−5.4 (− 11.1 to 0.0)	−2.7 (− 8.2 to 2.4)	0.10
1 year	−21 (− 24 to − 11)	−8 (− 16 to 0)	**0.002**
2 years	−18 (− 23 to − 7)	−8 (− 17 to 0)	0.09
3 years	−12 (− 14 to − 4)	−6 (− 16 to 0)	0.50
4 years	−13 (− 24 to 0)	−13 (− 16 to 0)	0.60
5 years	−13 (− 23 to − 4)	−16 (− 16 to − 6)	0.60
HerQLes Summary Score, median (IQR)			
Baseline	40 (22–62)	45 (25–65)	0.40
30 days	46 (30–87)	58 (37–87)	0.20
1 year	92 (76–97)	80 (63–95)	0.30
2 years	93 (88–98)	82 (50–93)	**0.01**
3 years	78 (45–92)	80 (68–100)	0.50
4 years	87 (82–93)	88 (72–93)	0.90
5 years	92 (83–96)	100 (85–100)	0.50

*PROMIS* Patient-Reported Outcomes Measurement Information System, *HerQLes* Hernia-Related Quality of Life Survey, *SD* standard deviation. Bold p-values indicate statistical significance (*p* < 0.05). Available sample sizes at each follow-up interval: 30 days *n* = 264, 1 year *n* = 75, 2 years *n* = 54–56, 3 years *n* = 41–42, 4 years *n* = 26, 5 years *n* = 14

### Sensitivity analysis

To address potential concern regarding outcome ascertainment in the primary analysis, a pre-specified sensitivity analysis was conducted restricting the cohort to patients with available one-year PROMIS Pain 3a follow-up data (*n* = 3,459; 21 emergent, 3,438 elective). In this follow-up-enriched cohort, PSM at a 1:2 ratio yielded a matched cohort of 60 patients (20 emergent, 40 elective). The primary finding was consistent with the main analysis: emergent patients reported significantly lower PROMIS Pain 3a T-scores at one year (median 31 [IQR 31–44] vs. 42 [IQR 31–52], *p* = 0.01). Notably, the pain advantage was sustained through two years in the sensitivity cohort (median 31 [IQR 31–36] vs. 46 [IQR 37–52], *p* = 0.002), with convergence by year three. HerQLes scores similarly favored emergent patients at two years (median 92 [IQR 85–97] vs. 63 [IQR 41–83], *p* = 0.002) and four years (median 92 [IQR 82–93] vs. 60 [IQR 43–78], *p* = 0.03). Complication rates and pragmatic recurrence were equivalent between matched groups at all time points (Supplementary Table 1).

## Discussion

In this propensity score matched analysis of 11,831 patients from the ACHQC registry, emergent VHR was associated with significantly lower PROMIS Pain 3a T-scores at one year compared to elective repair (median 31 [IQR 31–38] vs. 40 [IQR 31–46], *p* = 0.01), corresponding to a median improvement from baseline of − 21 (IQR − 24 to − 11) versus − 8 (IQR − 16 to 0) T-score points (*p* = 0.002). This difference exceeded the estimated minimum clinically important difference (MCID) of 2–3 T-score points for this instrument in hernia populations, suggesting the observed improvement carries clinical as well as statistical significance [8, 12]. Importantly, 30-day pain scores, complication rates, and hernia recurrence rates were equivalent between matched groups at all time points, and the one-year pain advantage in emergent patients attenuated to non-significance by two years and beyond. These findings were directionally consistent and statistically robust in a sensitivity analysis restricted to patients with complete one-year follow-up data, in which the pain advantage was sustained through two years (Supplementary Table 1). Contrary to our hypothesis and conventional clinical assumption, these findings suggest that emergent VHR does not result in worse patient-reported pain outcomes relative to well-matched elective controls [1, 2]. 

The existing literature on PROMIS Pain 3a outcomes after VHR is derived almost exclusively from elective repair populations, providing important benchmarks against which our findings can be contextualized [3–6, 8, 9, 13, 14]. Randomized controlled trials, including Krpata et al. comparing mesh weights and Ellis et al. comparing transfascial fixation approaches, reported median PROMIS Pain 3a T-scores in the range of 46 at 30 days after open retromuscular elective repair, with no significant differences between intervention arms at any time point [8, 9]. Alzatari et al. and Remulla et al. registry-based studies corroborate this trajectory, with both reporting median one-year PROMIS Pain 3a scores of approximately 31 in elective cohorts regardless of analgesic regimen or operative approach, consistent with the one-year scores observed in our matched elective group [6, 13]. Sandø et al., in a systematic review of long-term PROs after elective VHR, noted that clinically relevant pain improvement was observed primarily in patients with large incisional hernias, underscoring the heterogeneity of pain recovery even within elective populations and providing further context for the more pronounced improvement seen in our emergent cohort [14]. 

Despite the breadth of this literature, Shen et al., in a scoping review of 118 randomized controlled trials of VHR, found that no study stratified pain outcomes by repair acuity, confirming the evidence gap this study addresses [15]. The closest available acuity-stratified data come from Kim and Towfigh, who analyzed over 73,000 ACHQC cases and found that early quality-of-life improvement, measured by HerQLes rather than PROMIS, was most pronounced among higher-acuity emergent repairs, a directionally consistent finding with our results, though the instruments and populations differ [1]. Ali et al., in a Swedish population-based registry study of over 2,000 primary ventral hernia repairs including both elective and emergency cases, found no significant association between emergency surgery and postoperative pain after multivariable adjustment, though pain was assessed as a binary variable rather than with a validated patient-reported outcome instrument [7]. 

The mechanism underlying the superior one-year pain trajectory in emergent patients is not directly ascertainable from registry data but warrants consideration. One plausible explanation is the contrast effect, emergent patients present with substantially higher baseline pain burdens, driven by acute obstruction and peritoneal inflammation, and surgical resolution of the inciting pathology produces a more dramatic and perceptible reduction in pain compared to elective patients whose preoperative pain, though present, is typically more chronic and insidious [1, 2]. This phenomenon is consistent with findings from Hatchimonji et al., who prospectively evaluated PROMIS-29 domains in emergency general surgery patients and documented significantly elevated pain interference scores relative to population norms at the initial postoperative visit, suggesting that this population begins from a high pain state that surgery actively resolves [16]. Additionally, emergent patients who successfully recover and complete long-term follow-up may represent a selected, resilient cohort who tolerated high-acuity surgery without major complications, introducing survivorship bias that registry data cannot fully correct for even with rigorous propensity matching. The attenuation of the pain benefit by year two, with convergence of scores across both groups at levels well below the population mean of 50, is also noteworthy and may reflect that long-term pain equilibration after VHR is largely driven by hernia-specific and mesh-related factors rather than the acuity of the index operation [5, 6, 8, 9, 13]. 

The MCID estimates used in this study (2–3 T-score points) are derived from chronic pain populations and preliminary hernia registry data, as no formal hernia-specific MCID has been established for PROMIS Pain Intensity 3a. Vollert et al. reported MCID estimates of 1.2–1.6 on an 11-point scale for acute surgical pain, with higher baseline pain associated with higher MCID values (1.0 for mild pain, 2.1 for severe pain at rest), which may be particularly relevant to the emergent cohort in our study [8, 12, 17]. These findings have practical implications for surgical counseling, patients presenting emergently for VHR should not be informed to expect worse long-term pain outcomes than their elective counterparts, and surgeons operating in the emergent setting should be reassured that definitive repair, when feasible, does not appear to compromise patient-reported pain recovery [1, 2, 16]. 

## Limitations

This study has several important limitations. First, emergent VHR represented only 1% of the analytical cohort (*n* = 94), and despite a 1:3 matching ratio, the matched emergent group contained only 71 patients. This limits statistical power, particularly for extended follow-up intervals where available sample sizes were small and precludes definitive conclusions at two years and beyond. Second, although PSM was performed with 31 covariates including baseline PRO scores, residual confounding from unmeasured variables including surgeon experience, institutional resources available in the emergent setting, and perioperative pain management protocols cannot be excluded. Third, the ACHQC captures patients at participating institutions that have elected to engage in quality improvement efforts, which may not be representative of all centers performing emergent VHR in the United States.

Fourth, the MCID of 2–3 T-score points applied here was derived from hernia trial design parameters rather than a formally validated anchor-based threshold specific to this population, and no professional society has established a hernia-specific MCID for PROMIS Pain 3a to date [8, 12, 17]. Fifth, the HerQLes two-year finding favoring emergent patients, while statistically significant, was based on a small matched sample of 56 patients and should be interpreted with caution. Finally, follow-up attrition in the matched cohort was substantial. Only 75 of 264 matched patients (28%) had one-year PROMIS Pain 3a data available, and only 14 patients (5%) had five-year data. While this reflects the longitudinal follow-up structure of the ACHQC registry rather than active withdrawal, it limits the statistical reliability of outcomes beyond one year. Furthermore, whether emergent patients were systematically less likely to complete long-term follow-up than elective patients potentially introducing differential attrition bias cannot be assessed from registry data alone, and this possibility should be considered when interpreting the primary outcome.

## Conclusion

In conclusion, this propensity score matched analysis of ACHQC registry data demonstrates that emergent VHR is associated with superior patient-reported pain outcomes at one year compared to well-matched elective controls, with equivalent complication and recurrence rates. This finding challenges the prevailing assumption that emergent surgical circumstances portend worse pain recovery and provides actionable data for preoperative counseling in this population. Future studies with larger emergent cohorts, longer follow-up, and granular data on perioperative pain management protocols are needed to characterize the mechanisms underlying this trajectory and to define optimal care pathways for patients requiring urgent abdominal wall repair.

## Supplementary Information

Below is the link to the electronic supplementary material.

Supplementary Material 1

## Declarations

### Meeting presentation

This work was presented at the Society of American Gastrointestinal and Endoscopic Surgeons (SAGES) March 2026, Tampa, FL.
