# Supplementary material for: Pain outcomes in emergent versus elective ventral hernia repair: Propensity score matched analysis
Source: Hernia. 2026 Oct 1;30(1):367. doi: 10.1007/s10029-026-03842-0 (PMC13630787; doi:10.1007/s10029-026-03842-0)
Supplement: Supplementary file 1 [file 10029_2026_3842_MOESM1_ESM.docx]

**SUPPLEMENTARY TABLE 1. Sensitivity Analysis — Patients with Complete One-Year PROMIS Follow-up, Propensity Score Matched Cohort (Emergent n = 20, Elective n = 40)**

| Outcome | Emergent VHR (n = 20) | Elective VHR (n = 40) | P-value |
| --- | --- | --- | --- |
| Baseline Characteristics After Matching |  |  |  |
| Age, median (IQR) (years) | 60 (51–65) | 60 (52–66) | 0.81 |
| Female sex, n (%) | 10 (50%) | 23 (57%) | 0.62 |
| BMI, median (IQR) (kg/m²) | 37 (32–41) | 33 (28–39) | 0.22 |
| ASA Class 3–4, n (%) | 13 (65%) | 24 (60%) | 1.0 |
| Wound class contaminated/dirty, n (%) | 5 (25%) | 14 (35%) | 0.91 |
| PROMIS Pain 3a T-score at baseline, median (IQR) | 53 (49–58) | 56 (46–60) | 0.50 |
| HerQLes score at baseline, median (IQR) | 39 (25–55) | 40 (15–54) | 0.80 |
| PROMIS Pain 3a T-score, median (IQR) |  |  |  |
| Baseline | 53 (49–58) | 56 (46–60) | 0.50 |
| 30 days | 44 (31–52) | 46 (43–54) | 0.20 |
| 1 year | 31 (31–44) | 42 (31–52) | **0.01** |
| 2 years | 31 (31–36) | 46 (37–52) | **0.002** |
| 3 years | 40 (31–44) | 45 (32–51) | 0.20 |
| 4 years | 40 (31–44) | 46 (34–51) | 0.50 |
| 5 years | 37 (31–44) | 31 (31–46) | 1.0 |
| Change in PROMIS Pain 3a from Baseline, median (IQR) |  |  |  |
| 30 days | −7 (− 19 to − 3) | −6 (− 12 to 0) | 0.40 |
| 1 year | −21 (− 24 to − 11) | −13 (− 19 to − 6) | 0.06 |
| 2 years | −21 (− 24 to − 18) | −8 (− 18 to − 3) | **0.007** |
| 3 years | −15 (− 21 to − 6) | −12 (− 14 to − 6) | 0.50 |
| 4 years | −12 (− 23 to − 3) | −12 (− 15 to − 7) | 0.60 |
| 5 years | −14 (− 23 to − 9) | −13 (− 17 to − 10) | 0.70 |
| HerQLes Summary Score, median (IQR) |  |  |  |
| Baseline | 39 (25–55) | 40 (15–54) | 0.80 |
| 30 days | 52 (23–79) | 52 (20–77) | 0.80 |
| 1 year | 88 (77–97) | 82 (51–93) | 0.10 |
| 2 years | 92 (85–97) | 63 (41–83) | **0.002** |
| 3 years | 83 (52–92) | 53 (33–95) | 0.30 |
| 4 years | 92 (82–93) | 60 (43–78) | **0.03** |
| 5 years | 92 (85–92) | 60 (26–91) | 0.20 |
| 30-Day Surgical Outcomes |  |  |  |
| SSI, n (%) | 1 (5%) | 3 (8%) | 0.71 |
| SSO, n (%) | 1 (5%) | 2 (5%) | 1.0 |
| Readmission, n (%) | 2 (11%) | 5 (13%) | 0.81 |
| Reoperation, n (%) | 0 (0%) | 3 (8%) | 0.21 |
| Any complication, n (%) | 5 (25%) | 10 (25%) | 1.0 |
| Hernia Recurrence |  |  |  |
| Pragmatic recurrence at 1 year, n/N (%) | 3/20 (15%) | 9/40 (22%) | 0.50 |
| Pragmatic recurrence at 2 years, n/N (%) | 1/13 (8%) | 7/21 (33%) | 0.09 |
| Pragmatic recurrence within 5 years, n/N (%) | 3/20 (15%) | 14/40 (35%) | 0.10 |

*PROMIS, Patient-Reported Outcomes Measurement Information System; HerQLes, Hernia-Related Quality of Life Survey; IQR, interquartile range; SSI, surgical site infection; SSO, surgical site occurrence. Bold p-values indicate statistical significance (p < 0.05). Available sample sizes at each follow-up interval: 30 days n = 60, 1 year n = 60, 2 years n = 27, 3 years n = 23, 4 years n = 14, 5 years n = 13.*
